# Armoring Boron Nitride with Pyrolytic Carbon Layers for Tunable Rigidity and Flexibility

**DOI:** 10.1002/advs.202504649

**Published:** 2025-06-23

**Authors:** Meng Lan, Yu Pei, Kexin Huang, Yudong Zhou, Xiao Han, Qiangang Fu

**Affiliations:** ^1^ Shaanxi Key Laboratory of Fiber Reinforced Light Composite Materials Science and Technology on Thermostructural Composite Materials Laboratory Northwestern Polytechnical University Xi'an 710072 P. R. China; ^2^ School of Medicine Xi'an Jiaotong University Xi'an Shaan'xi Province 710061 P. R. China

**Keywords:** boron nitride, ceramic aerogels, customization, mechanical properties, pyrolytic carbon

## Abstract

Ceramic aerogels are acclaimed as ideal materials for extreme environments due to their ultrahigh porosity, low density, and exceptional thermal stability. However, the practical application of traditional ceramic aerogels is constrained by their inherent mechanical brittleness. Herein, a strategy is proposed to customize mechanically tunable aerogels by armoring ceramic nanounits with pyrolytic carbon (PyC). The PyC encapsulating layers with various thicknesses are transformed into ductile tights or rigid armors, facilitating the mechanical customization of BN@PyC aerogel from superelasticity to rigidity across scales. Interestingly, the PyC armor imparts unique versatility to the aerogel, including flame retardancy, elastic response conductivity, and thermal conductivity. Furthermore, owing to the thermal stability of the PyC armor, the BN@PyC aerogel maintains its mechanical integrity even after high‐temperature thermal treatment, and exhibits an unexpected resistance to butane torch ablation. Integrating mechanical stability, high‐temperature resistance, and multifunctionality, BN@PyC aerogels offer new possibilities for scalable applications in extreme environments. This facile strategy of armoring brittle ceramic aerogels with PyC provides a novel reference for customizing the mechanical properties of multifunctional aerogels.

## Graphical abstract

By precisely controlling the thickness of the PyC armor, the BN@PyC aerogel achieves a remarkable transformation in mechanical properties, spanning multiple orders of magnitude from flexibility to rigidity, while simultaneously exhibiting versatility, including elastic responsive conductivity, enhanced thermal conductivity, and outstanding ablative resistance.

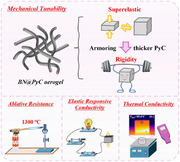

## 1 Introduction

From thermal protection of deep space probes to thermal management in supersonic vehicles, the demand for extreme materials has evolved from a singular focus on high‐temperature resistance to a comprehensive requirement for mechanical robustness, light‐weight and multifunctionality.^[^ ^1^, ^2^, ^3^ ^]^ Ceramic aerogels, characterized by their high porosity, low density, and exceptional thermal stability, have emerged as ideal candidates.^[^ ^4^, ^5^, ^6^, ^7^, ^8^ ^]^ However, traditional ceramic aerogels, composed of loosely cross‐linked nanoparticles, often struggle to withstand high mechanical loads, leading to unexpected failures.^[^ ^9^, ^10^, ^11^ ^]^ To address this inherent weakness, researchers have been exploring new strategies, such as introducing a secondary phase to support the network structure or replacing the building blocks of ceramic aerogels.^[^ ^12^, ^13^ ^]^ Notably, constructing aerogel networks using ceramic nanostructures with high aspect ratios has proven to be an effective approach for significantly enhancing their mechanical stability.^[^ ^14^, ^15^ ^]^

Boron nitride (BN) aerogels, composed of BN nanounits, offer additional options for various applications under extreme conditions due to their unique chemical stability and high‐temperature oxidation resistance.^[^ ^16^, ^17^, ^18^ ^]^ However, their intrinsic properties limit their thermal stability to a maximum of 900 °C, which restricts their application in extreme conditions.^[^ ^19^ ^]^ In addition, BN aerogels suffer from poor mechanical strength and irreversible deformation after cyclic compression due to their low structural cross‐linking density.^[^ ^20^ ^]^ To overcome these challenges, heterogeneous element doping such as the intercalation of Ca atom and high‐temperature ceramic phase introduction via template‐assisted approach have been adopted.^[^ ^21^, ^22^ ^]^ However, the complex fabrication process and high production costs hinder their scalability. In addition, achieving a synergistic enhancement of mechanical performance and thermal protection capabilities in BN aerogels remains a critical and unresolved challenge.

Pyrolytic carbon (PyC), composed of locally ordered graphene‐like fragments in a layered configuration, could perform as the ideal outer armor for enhancing mechanical properties and thermal protection due to its exceptional strength, ablation resistance, and thermal conductivity.^[^ ^23^, ^24^, ^25^ ^]^ To be specific, several recent investigations have demonstrated the effectiveness of PyC coatings on Al_2_O_3_ and Si_3_N_4_ aerogels to improve the load bearing capability via strengthened interlocking between building blocks.^[^ ^26^, ^27^ ^]^ Moreover, PyC has been extensively employed as an ablation‐resistant thermal protection layer in lightweight carbon/carbon composites, further showcasing its reliability.^[^ ^28^, ^29^, ^30^ ^]^ These findings collectively underscore the potential of PyC in developing mechanically tunable and thermally robust protective systems, motivating further exploration into scalable applications.

Herein, taking advantages of PyC in both mechanical and thermal performance modulation, we present an innovative design strategy to fabricate mechanically tunable ceramic aerogels through the integration of controllable PyC armor on assembled ceramic nanostructures. Employing chemical vapor deposition (CVD), we successfully synthesized BN@PyC aerogels with PyC armor of controlled thickness, allowing for a wide range of mechanical tunability from superelasticity to rigidity. Ultrathin PyC tights confers exceptional superelasticity to the aerogels, surpassing the performance of conventional inorganic aerogels. Notably, the thickness of the PyC armor and the formation of PyC welding joints are critical factors governing the transition from elastic to rigid behavior. The 3D network of PyC welded joints collaborates with the PyC armor thus to enhance the compressive strength significantly. In addition to the intrinsic flame retardancy, electrical conductivity, and thermal conductivity, the PyC armor imparts outstanding mechanical stability to BN@PyC aerogels even after thermal treatment. Remarkably, the PyC thermal protection armor effectively shields the BN aerogel from structural damage under butane flame ablation at 1300 °C, significantly extending its operational temperature limit and demonstrating its potential for extreme thermal environments.

## 2 Results and Discussion

**Figure 1:**
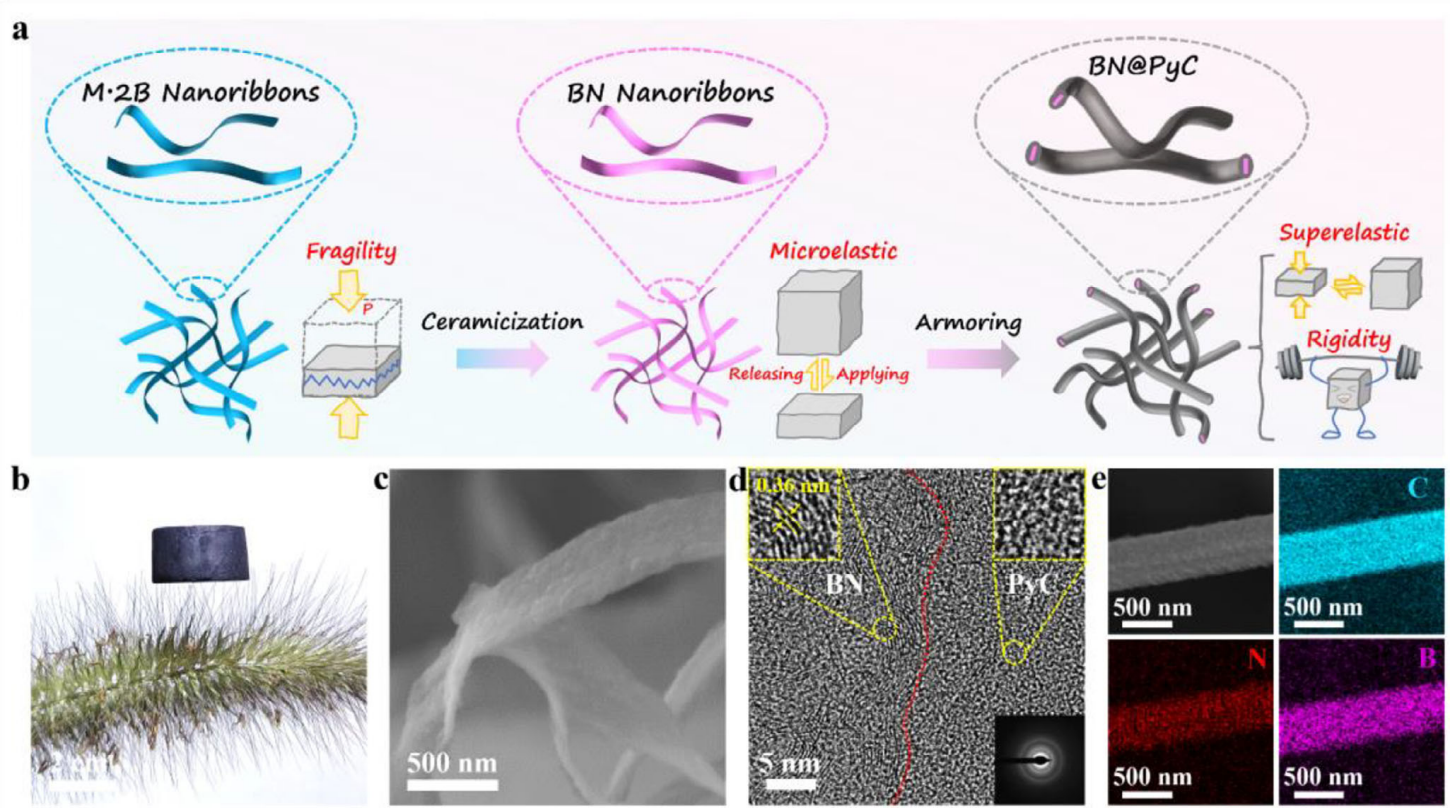
Schematic and general characterizations of the mechanically tunable BN@PyC aerogel. a) Preparation process. b) The photograph of lightweight BN@PyC aerogel on foxtail grass. c) The SEM image of BN nanoribbons armored by PyC exhibiting great flexibility with bending angles <90°. d) The HR‐TEM image showing the interface between the PyC layer and BN core along with the SAED pattern and enlarged lattice fringes. e) SEM and elemental mapping images of a single BN@PyC nanoribbon.

Through the strategic armoring of BN aerogel with PyC, mechanically tunable BN@PyC aerogels were successfully fabricated. As illustrated in **Figure** **1**a, the BN aerogel is synthesized via thermal treatment of the M·2B precursor. The M·2B aerogel features a 3D random network architecture, formed through the self‐assembly of 2D supramolecular ribbons, which serves as a structural template for the formation of BN nanobelts during pyrolysis. However, the hydrogen bond‐dominated supramolecular network imparts intrinsic brittleness to the M·2B aerogel, rendering it susceptible to structural collapse under external stress. In contrast, due to the flexible structural units constructed from sp^2^‐hybridized B‐N covalent networks, the BN aerogel exhibits a modest level of compressive resilience. Notably, the mechanical properties of the aerogel could be enhanced significantly after being armored with PyC layer. Moreover, a broad spectrum of mechanical properties can be tailored by tuning the thickness of the armor, facilitating a transition from superelastic to pressure‐resistant characteristics. The thickness of the PyC armor can be adjusted by modulating the deposition time. The BN@PyC aerogels labeled as BC_1_, BC_2_, BC_3_, and BC_4_ correspond to PyC deposition time of 0.125, 0.25, 0.5, and 2 h, respectively.

In addition, compared to the original lightweight BN aerogel (Figure S1, Supporting Information), the BN@PyC aerogel still maintains its inherent ultra‐lightweight feature with density as low as 30 mg cm^−3^, enabling it to rest effortlessly on a piece of foxtail grass (Figure 1b). Figure 1c showcases the bendable nanoribbon unit, which exhibits ribbon‐like flexibility with folding angles <90°. The integration of the PyC armor with the BN is tightly bonded, as further confirmed by HR‐TEM in Figure 1d. The boundary layer between PyC and BN aligns perfectly without any transitional layer. Notably, the measured interlayer spacing of 0.36 nm in BN significantly exceeds the 0.34 nm characteristic of standard hexagonal boron nitride (h‐BN), demonstrating turbostratic phase structure. Enlarged TEM images reveal the locally ordered layered structure of the BN alongside the disordered structure of PyC. Furthermore, the energy‐dispersive spectroscopy (EDS) elemental distribution in Figure 1e illustrates the uniform deposition of PyC on the BN surface.

**Figure 2:**
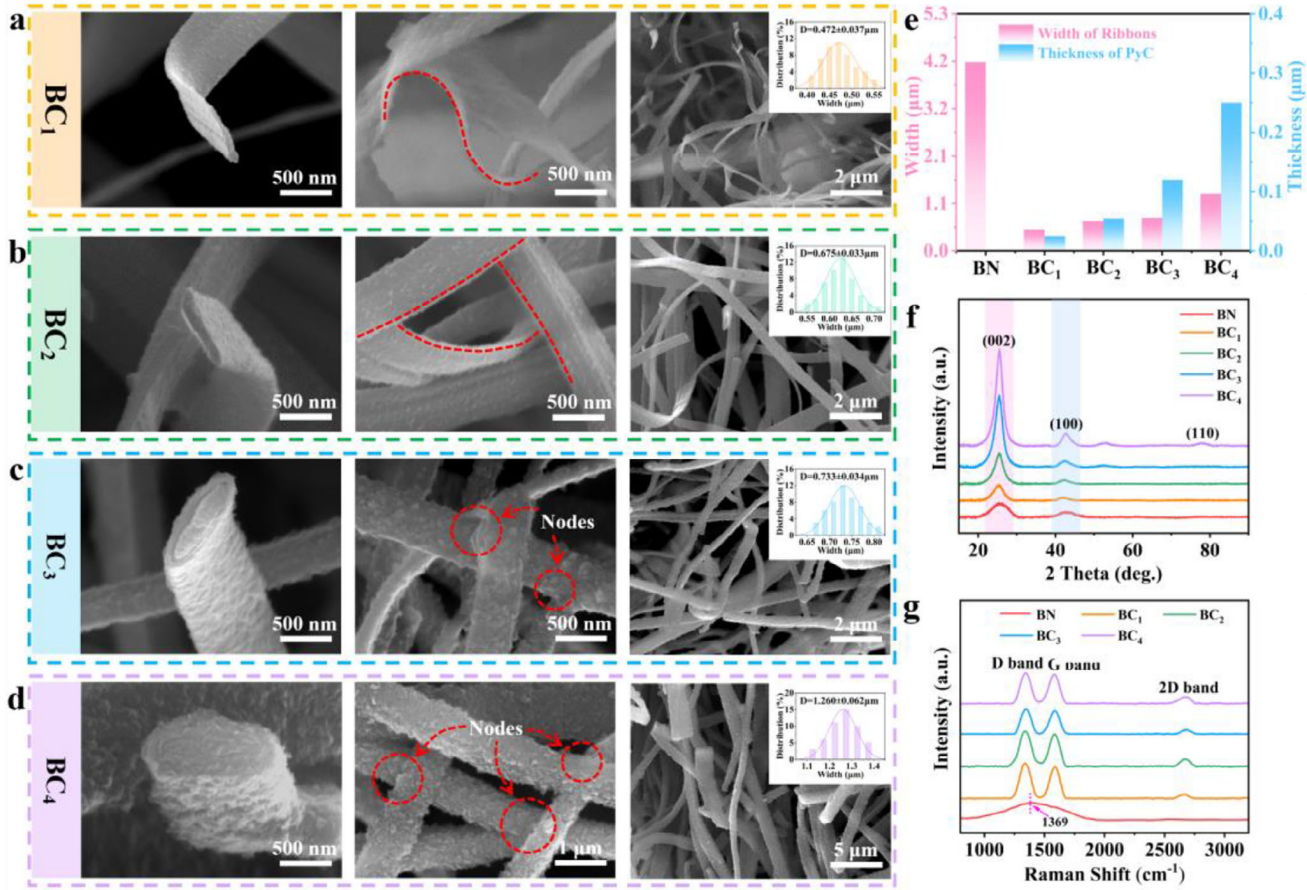
Characterizations of the BN@PyC aerogel with controllable armoring thickness. a–d) SEM images of BC_x_ (x = 1–4, the larger number represents a greater thickness of the depositing PyC layer). The inset presents the histogram distribution of the ribbon width. e) Variations in the thickness of PyC layer and width of BN@PyC ribbons. f) XRD patterns. g) Raman spectra.

By precisely controlling the thickness of the PyC armor (determined via EDS line scanning, Figure S2, Supporting Information), the 3D network structure of BN@PyC aerogels achieves a multi‐stage transition from a flexible and resilient state to a rigid and pressure‐resistant form. This dynamic evolution of mechanical behavior is thoroughly validated by both microscopic morphology and crystallographic features (**Figure** **2**). Specifically, Figure 2a displays that after being armored with a 30 nm thick layer of PyC, the single nanoribbon of BC_1_ can bend at small angles (<90°), thus retaining the inherent flexibility of BN while exhibiting the toughness of PyC. The constructed 3D network is randomly interwoven without nodes. As the thickness of PyC increases to 60 nm, the single nanoribbon of BC_2_ retains the ability to bend at larger angles (>90°), while the 3D network remains a single interwoven structure without any crossover nodes (Figure 2b). When the thickness of PyC is further doubled to 120 nm, the connections between the ribbons become tighter, forming nodes through PyC welding. The single ribbon loses its flexibility, resulting in a rigid 3D network. Once the thickness of PyC exceeds 250 nm, the number of welding points between the ribbons increases, with nodes connecting at least three ribbons. Consequently, the 3D network becomes fully armored with PyC, forming a pressure‐resistant rigid structure. Figure 2e illustrates that as the deposition thickness of PyC increases, the width of individual ribbons gradually expands. Interestingly, all PyC‐armored samples exhibit significantly narrower widths than pristine BN nanoribbons. This might be attributed to BN crystallinity‐driven contraction induced by high‐temperature treatment, supported by the substantial width reduction shown in Figure S4 (Supporting Information). The enhanced crystallinity is further confirmed by XRD (Figure S5, Supporting Information).^[^ ^31^ ^]^

XRD patterns in Figure 2f indicate that the original BN exhibits small and broad characteristic peaks. The peaks at 25.6° and 41.5° correspond to the (002) and (100) lattice planes of h‐BN, respectively.^[^ ^32^, ^33^ ^]^ As the thickness of the PyC armor increases, BC samples exhibit progressive intensification of the (002) diffraction peak accompanied by significant reduction in full width at half maximum (FWHM), reflecting increase of crystallite size and enhanced structural ordering within the PyC layer.^[^ ^34^, ^35^ ^]^ In the Raman spectra shown in Figure 2g, a broad peak appears at 1369 cm^−1^ for BN, attributed to the E_2g_ phonon mode, corresponding to the characteristic peak of h‐BN.^[^ ^36^, ^37^ ^]^ The BC samples present characteristic peaks at 1350 and 1580 cm^−1^, corresponding to the D band and G band, respectively. The D band primarily results from the disturbance of sp^2^ hybridized carbon atoms within the hexagonal lattice, which is linked to defects in the graphite structure, while the G band is attributed to the elongation and contraction of bonds between sp^2^ hybridized carbon atoms, reflecting the degree of graphitization.^[^ ^38^, ^39^ ^]^ Notably, all samples reveal high intensity in the D band, indicating the presence of defects associated with disordered carbon. As the deposition time increases, the I_D_/I_G_ ratio of BC samples continuously decreases from 1.11 to 1.05, directly confirming the enlargement of aromatic planes and reduced in‐plane defect density, aligning with the XRD results.^[^ ^40^, ^41^ ^]^

**Figure 3:**
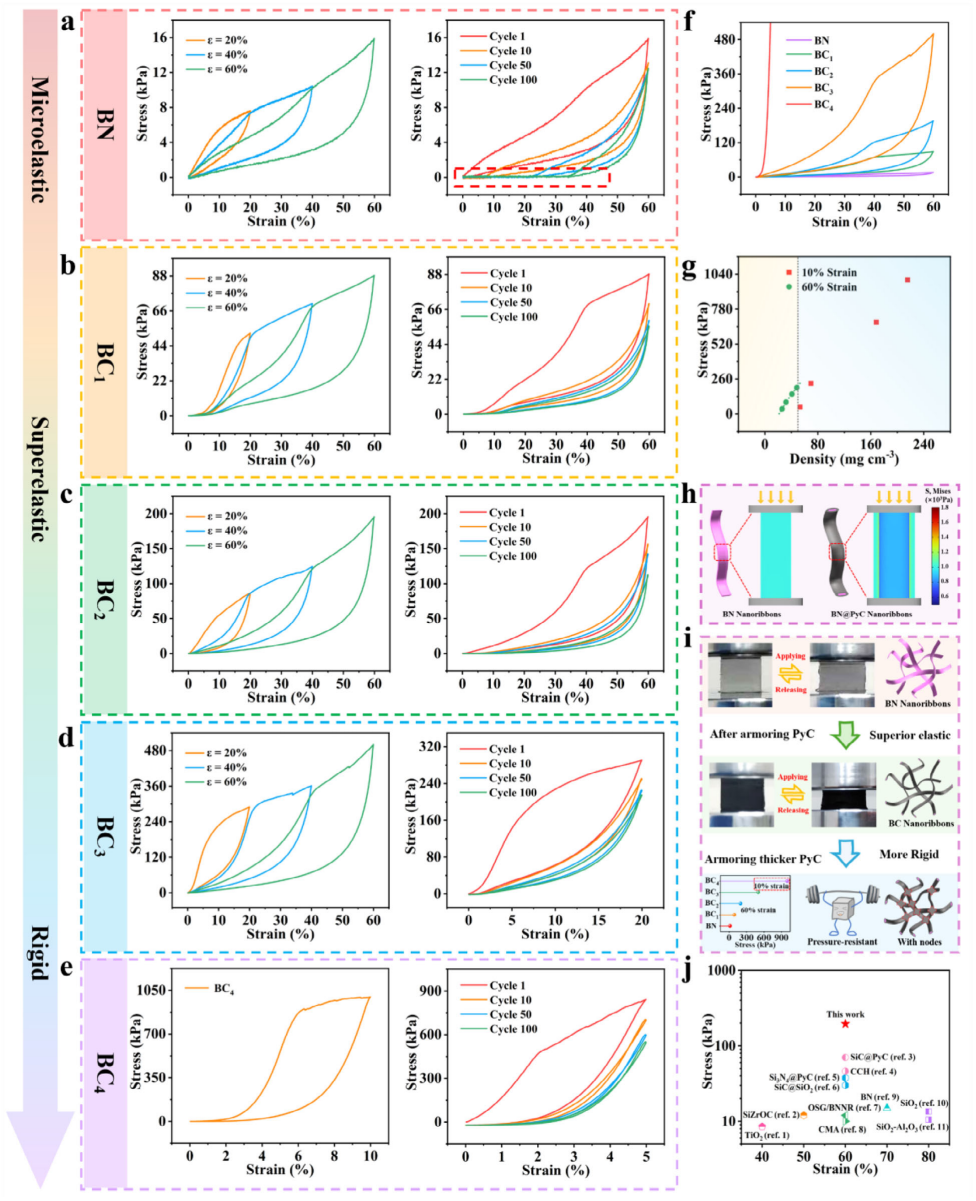
Tunable mechanical performance for BN@PyC aerogel. a–e) Compressive stress‐strain curves and compression fatigue tests for BN and BC_x_ samples. f) Comparison of the compressive stress‐strain curves of different samples. g) Dependency of the compressive strength for BC samples with density. h) Finite element simulation of stress distribution for in a single nanoribbon of BN and BN@PyC under compressive stress. i) Schematic diagram of the macro‐ and micro‐ structural evolutions. j) Maximum recoverable strain and ultimate stress of BC_2_ compared to other reported elastic ceramic samples.

The compression test was employed to investigate the impact of PyC armor on the mechanical properties of BN aerogels. As shown in **Figure** **3**a, the original BN aerogel exhibits a compressive strength of only 15.9 kPa at 60% strain, with a permanent deformation of 32% after 100 compression cycles. Its initial energy loss coefficient reaches as high as 0.47 (Figure S6, Supporting Information). Surprisingly, the ultra‐thin PyC armor (30–60 nm), resembling a bodysuit, imparts superelasticity to the BC aerogel. As shown in Figure 3b, BC_1_, equipped with ultra‐thin PyC armor (30 nm), maintains a compressive strength of 55.7 kPa and a Young's modulus of 92.3 kPa after 100 compression cycles at 60% strain (Figure S7, Supporting Information). In Figure 3c, prior to the formation of welding points, the increase in PyC armor thickness to 60 nm optimizes the compressive elasticity of BC_2_ due to the sliding characteristics and high toughness of PyC. This performance far exceeds that of similar inorganic elastic aerogels (Figure 3j), with a maximum compressive strength of 195.9 kPa at 60% strain.^[^ ^27^, ^42^, ^43^, ^44^, ^45^, ^46^, ^47^, ^48^, ^49^, ^50^, ^51^ ^]^ After 100 compression cycles, the energy loss coefficient stabilizes at 0.36 (Figure S8, Supporting Information). When the PyC armor thickness exceeds 120 nm, fixed 3D network welding points are formed, and the rigidity of the aerogel is gradually enhanced. As shown in Figure 3d, the rigid armor enables BC_3_ to achieve a compressive strength of 500 kPa at 60% strain. Even under a lower strain of 20% after cyclic loading, it retains a compressive strength of 215.5 kPa, with the Young's modulus exceeding 1 GPa (Figure S9, Supporting Information). Figure 3e demonstrates that as the number of PyC welding points increases, BC_4_ exhibits exceptional compressive rigidity under a low strain of 10%, achieving a compressive strength of 998 kPa.

Figure 3f vividly illustrates that tuning the PyC armor thickness enables cross‐scale mechanical customization of the aerogel. As the thickness of the PyC armor increases, the density of the BC samples rises, leading to enhanced compressive strength. With the formation of welding points, the superelastic properties gradually transition to rigidity. Figure 3g reveals a strong linear correlation (R^2^ = 0.995) between density and compressive strength at 60% strain for superelastic samples with densities below 50 mg cm^−3^. For rigid samples with densities exceeding 60 mg cm^−3^, the density of PyC welding nodes increases sharply, forming a continuous network, and compressive strength under 10% strain remains linearly correlated (R^2^ = 0.99). Furthermore, a logarithmic‐linear relationship is observed between the compressive modulus and PyC thickness (R^2^ = 0.99), highlighting the critical role of the core‐shell structure thickness in governing mechanical performance (Figure S11, Supporting Information).

The finite element simulation in Figure 3h explains the critical role of PyC armor in enhancing toughness. Under compressive stress applied from above, the sliding characteristics of the PyC armor significantly reduce the peak von Mises stress of the BN@PyC core‐shell structure.^[^ ^26^, ^40^ ^]^ Stress distribution transitions from localized concentration to multidirectional dispersion. Figure 3i highlights that cross‐scale mechanical customization of aerogels is closely associated with the structural design of the fundamental load‐bearing units. Figure S12 (Supporting Information) illustrates the stress evolution in different armored structures. As the PyC armor thickness increases, the initial enhancement of stress dispersion reduces the peak stress in the BN core, shifting the stress distribution from axial concentration to radial multi‐point dissipation. A critical transition occurs when the thickening forms welded joints, transforming the structure from discrete core‐shell units into an interconnected 3D rigid network. The welded nodes eliminate stress concentration at contact points, reconstructing load transfer paths to redistribute stress through networked shear deformation (Figure S13, Supporting Information). Figure S14 (Supporting Information) demonstrates that increasing the number of nodes significantly improved stress distribution homogeneity. This shift from sliding dissipation at flexible armor interfaces to collaborative load‐bearing in the rigid welded network directly triggers a significant modulus leap, manifesting as characteristic serrated fluctuations on the stress‐strain curve. Essentially, the aerogel's superelasticity originates from the synergy between interfacial sliding dissipation and stress redistribution in PyC armor, while its rigidity and ultrahigh modulus stem from the collaborative load‐bearing mechanism of the 3D rigid network constructed by PyC welded nodes during multi‐directional stress transfer. This mechanical reinforcement mechanism, achieved through interfacial armor for sliding energy dissipation and network load‐bearing, establishes a novel paradigm for the synergistic enhancement of both high strength and high modulus.

**Figure 4:**
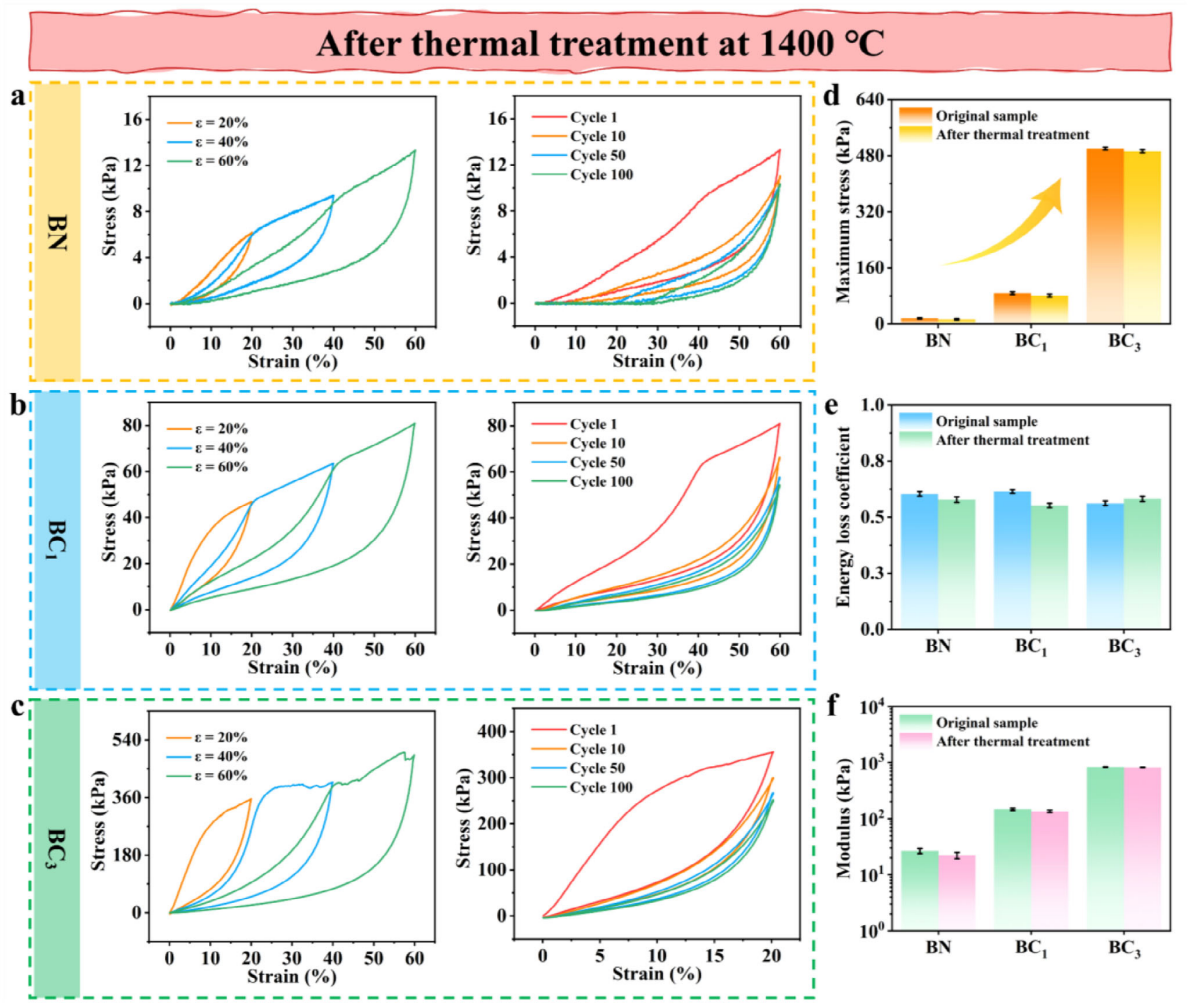
Thermally robust mechanical performance for BN@PyC aerogel. a–c) Compressive stress‐strain curves and compression fatigue tests of BN and BC samples after heat treatment at 1400 °C in an argon atmosphere. d–f) Comparisons of the mechanical properties before and after heat treatment, including d) maximum compressive strength at 60% deformation, e) energy loss coefficient, and f) Young's modulus.

To assess the high‐temperature mechanical stability of the aerogel, the mechanical compressive properties of BN@PyC were evaluated after being heat‐treated at 1400 °C for 3 h in an argon atmosphere. As shown in **Figure** **4**a, the compressive strength of heat‐treated BN decreased by 16.9% at strain of 60%, attributed to the reduction in the bandwidth of BN stress‐bearing units after high‐temperature treatment. When a bending moment is applied, the maximum stress is inversely proportional to the elastic cross‐sectional area, meaning that the reduced bandwidth leads to a corresponding decrease in compressive strength and modulus.^[^ ^52^ ^]^ After 100 compression cycles, the compressive strength further declined to 10.2 kPa (Figure S15, Supporting Information).

PyC demonstrates superior thermal stability than BN in inert high‐temperature environments due to robust covalent bonding and partially ordered structure.^[^ ^23^, ^24^ ^]^ As a consequence, even after heat treatment, BN@PyC aerogels retained remarkable elasticity and rigidity. As depicted in Figure 4b, owing to the high‐temperature oxidation resistance protection provided by the PyC armor, the superelastic BC_1_ sample maintains a compressive strength of 80 kPa under a strain of 60%, with the Young's modulus only decreasing by 2.4% (Figure S16, Supporting Information). This outcome demonstrates that the PyC armor not only effectively safeguards the structural integrity of BN but also remarkably enhances its mechanical properties in high‐temperature environments. Specifically, the 3D network constructed by the PyC welding joints and nanoribbons not only optimizes the load transfer pathway but also inhibits interlayer delamination at high temperatures. Figure 4c illustrates that the rigid BC_3_ sample exhibits similar mechanical degradation under high strain, with its compressive strength decreasing by merely 5.9% under a strain of 60%.

In addition, the compressive strength under a strain of 60% increases exponentially with the increase in the thickness of the PyC armor (Figure 4d). The difference before and after the heat treatment is negligible and falls within the error range, which can be attributed to the high strength property of PyC at high temperature. Notably, in Figure 4e, the energy dissipation coefficient of the BC_3_ sample increases significantly, which is directly related to the suppression of interfacial slippage by the PyC rigid network. Figure 4f reveals the order‐of‐magnitude changes in the Young's modulus among the samples, further validating the controllability of the PyC armor over the high‐temperature mechanical properties across different magnitudes. The BN@PyC aerogels exhibit outstanding mechanical stability in an inert high‐temperature environment, highlighting their immense potential in aerospace applications.

**Figure 5:**
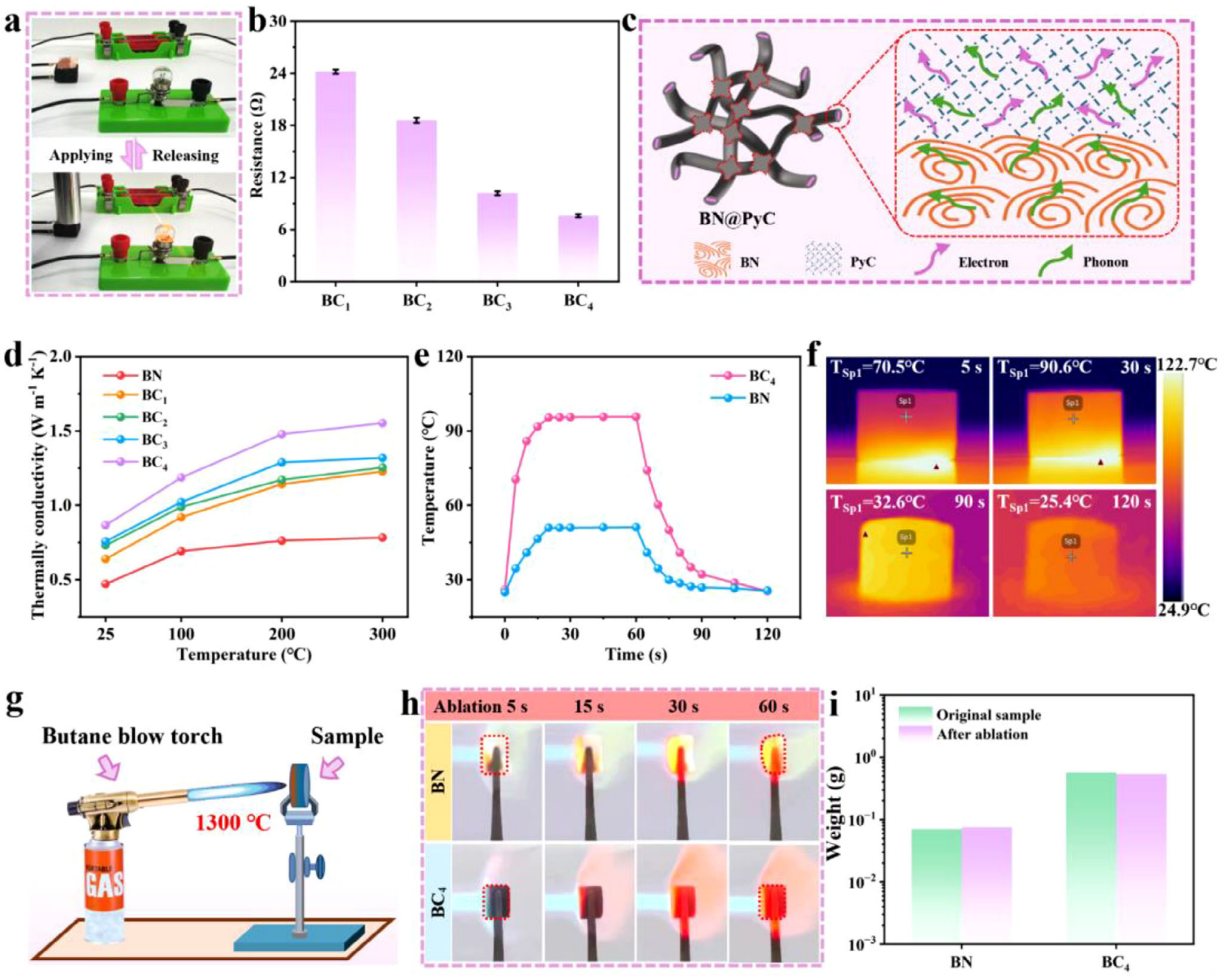
Multifunctionality of BN@PyC aerogel. a) Variation in brightness of bulb during compression and release of BC. b) Electrical resistance for various BC samples. c) Schematic diagram of the thermal conduction mechanism of BN@PyC. d) Thermal conductivity of BN armored with PyC of varying thicknesses. e) Temperature variation curves of BN and BC_4_ on a 120 °C heating stage. f) Infrared thermal imaging of the temperature evolutions for BC during heating and cooling processes. g) Schematic diagram of the ablation under a butane torch flame. h) Optical photographs of the ablation for BN and BC and i) changes in mass before and after ablation.

Building upon the precise mechanical tunability, the PyC armor further endows BN@PyC aerogels with unique multifunctionality (**Figure** **5**). By constructing a synergistic network of electrical conductivity, thermal management, and ablation resistance, the material exhibits multidimensional performance advantages under extreme conditions, laying the foundation for applications in intelligent sensing and thermal management. Figure 5a vividly illustrates the behavior of the highly elastic BC_1_ under external compressive forces. As the randomly oriented 3D network is compressed, the contact points between the outer PyC layers significantly increase, thereby forming conductive pathways that illuminate a light bulb. As demonstrated in Movie S1, the bulb periodically lights up and dims in response to cyclic compression of the sample, validating the rebound stability of the BN@PyC aerogel's 3D network while highlighting its potential as a stress sensor.

To determine the electrical conductivity of the aerogel quantitatively, a series of BC samples were measured, as shown in Figure 5b. As the PyC armor thickness increases, resistivity decreases significantly, indicating enhanced conductivity, which provides critical evidence for an electron‐based heat transfer mechanism and potentials. The optimization of thermal management performance further expands the application scenarios of BN@PyC, in which the PyC armor enhances thermal conductivity through a dual mechanism (Figure 5c). Specifically, the internal BN primarily conducts heat through phonons, while the matching armor of PyC and BN reduces the interfacial thermal resistance between them. Additionally, the conductive PyC welding points in the thermal network enhance the thermal conduction pathways through electron conduction, resulting in a significant increase in the thermal conductivity of the samples armored via PyC.^[^ ^53^, ^54^ ^]^

As depicted in Figure 5d, the thermal conductivity (k) of BN aerogel at room temperature is 0.472 W/(m·K). With the addition of PyC armor, the thermal conductivity of BC samples increases by up to 100%. As the temperature rises, the reduction in phonon relaxation time leads to a general increase in k across all samples. Notably, BC_4_ with the highest density of PyC welding points, achieves a thermal conductivity of 1.55 W/(m·K) at 300 °C, doubling its value at room temperature.

To evaluate the thermal performance visually, all samples were placed on a 120 °C heating platform, and temperature variations across their cross‐sections were recorded using an infrared thermal imager. Compared to BN aerogel, the PyC‐armored BC_1_ sample exhibited a minimum temperature increase of 27.9%, while the highly conductive BC_4_ sample reached a maximum temperature of 95.7 °C. Furthermore, as shown in Figure S20 (Supporting Information), higher k values correspond to faster heating rates, with each sample's heating trend aligning closely with its respective k value (Figure S21, Supporting Information). As depicted in Figure 5e, the thermally conductive BC_4_ rapidly reaches a steady‐state temperature within 30 s and cools down to 32.6 °C within 30 s after the heating platform is removed. The detailed macroscopic temperature changes are presented in Figure 5f. The behavior of rapid heating and cooling confirms that PyC armor significantly enhances the sensitivity and efficiency of thermal conduction.

Apart from electron and heat conduction functionalities, the protective effect of PyC armor is particularly significant in extreme thermal environments. As shown in Figure S22 (Supporting Information), owing to the inert nature of ceramic and carbon materials, both BN aerogel and BN@PyC aerogel remain stable without deformation, combustion, or other physicochemical changes when exposed to a 600 °C alcohol flame for 180 s. To investigate the protective limits of PyC armor, ablation resistance tests were conducted by exposing the samples to a 1300 °C butane torch flame, as illustrated in Figure 5g. Under the intense erosion of the butane flame, the edges of the BN sample showed significant burning and retreat. In contrast, the BC_4_ sample, protected by the PyC armor, withstood exposure to the butane flame for 60 s without apparent deformation (Figure 5h). Macroscopic optical images of the samples before and after ablation are provided in Figure S23 (Supporting Information). As shown in Figure 5i, both samples exhibited slight changes in mass due to surface oxidation processes. The BN sample exhibited structural degradation due to high‐temperature ablation, while showing a slight weight gain caused by oxide formation and carbon phase deposition. In contrast, only surface oxidation occurred on the pyrolytic carbon layer of the BC sample, with the underlying BN@PyC nanobelt network largely retaining its structural integrity. This conclusion corresponds well with the morphological evolution characteristics observed in the ablation zones (Figure S24, Supporting Information). Benefiting from the PyC armor, the BN@PyC sample demonstrated excellent stability and durability even at elevated temperatures. By integrating the cross‐scale design of conductive network reconstruction, thermal conductivity path optimization, and ablation‐resistant barrier construction, BN@PyC aerogel achieves a synergistic enhancement of mechanical‐electrical‐thermal‐ablation‐resistant properties. This multifunctional integrated property enables it unique advantages in the fields of intelligent thermal protection system for spacecraft and high‐temperature strain sensing, providing a paradigm for the design of the next generation of materials for extreme environments.

## 3 Conclusion

In summary, we propose a simple strategy to achieve cross‐scale tunability of the mechanical properties for ceramic aerogels by adjusting the thickness of the PyC armor. Thin‐layer PyC armor, acting as a “nanoscale tight suit”, imparts exceptional superelasticity to the aerogel, achieving a compressive strength of 195.9 kPa at 60% strain, while simultaneously enabling strain‐responsive electrical conductivity. As the PyC armor thickens and welded nodes form, it transforms into a rigid framework, increasing compressive strength by two orders of magnitude to reach 998 kPa at 10% strain. Furthermore, the PyC armor imparts additional functional properties to the aerogel, including exceptional high‐temperature mechanical stability, flame retardancy, ablation resistance, and enhanced thermal conductivity. This study provides a novel strategy for the precise tuning of the mechanical properties of ceramic aerogels, opening new avenues for their exploration in multifunctional applications, thereby showcasing their tremendous potential in fields such as aerospace, flame‐retardant materials, and flexible sensors.

## 4 Experimental Section

### Fabrication of BN@PyC

First, melamine (C_3_N_6_H_6_) and boric acid (H_3_B_3_O) in a molar ratio of 2:1 were added to a mixed solution of tert‐butanol and deionized water. The mixture was magnetically stirred in a water bath at 85 °C until the solution became transparent. Subsequently, the solution was allowed to cool naturally to room temperature, yielding a gel‐like substance. The melamine diborate (M·2B) precursor was obtained by freezing the resulting material at −20 °C for 24 h, followed by sublimation drying in a freeze dryer. Finally, the BN aerogel was obtained by subjecting the precursor to high‐temperature treatment (1400 °C) for 3 h in a flowing nitrogen atmosphere.

During the CVD process for PyC, the BN aerogel was placed in the heating zone of a tube furnace, maintained at atmospheric pressure, while the entire process was conducted in a flowing argon atmosphere. After heating to the target temperature at a rate of 5 °C/min, methane gas was introduced, where the flow rate of methane was 500 standard cubic centimeters per minute (sccm). The temperature was kept for 0.125 to 2 h to obtain BN@PyC samples (BC_x_, x = 1, 2, 3, 4).

### Characterization

The microstructure and elemental distribution of the samples were observed via a field emission scanning electron microscope (SEM, TESCAN MIRA3). Electronic diffraction patterns were further analyzed with a transmission electron microscope (TEM, FEI Talos F200X). The phase composition was determined through X‐ray diffraction (XRD, Bruker D8 DISCOVER A25, Cu Kα‐radiation) and Raman spectroscopy (InVia, Renishaw, a 532 nm He‐Ne laser). The mechanical compression tests were conducted on an electronic universal testing machine (CMT5304) equipped with a 2000 N load cell, applying a compression rate of 5 mm min^−1^ for standard tests and 10 mm min^−1^ for cyclic tests. Thermal conductivity was assessed with a laser flash apparatus (Netzsch LFA 427 Microflash). The temperature changes of the samples on the heating platform (JF946‐100) were recorded by an infrared thermal imaging camera (FOTRIC V7). The ablation resistance tests of the samples were performed by a butane torch.

### Finite Element (FE) Simulation

Finite Element (FE) simulation was performed based on COMSOL Multiphysics 6.2 software, employing stressed elements with different structures to calculate the corresponding von Mises stress distribution. The width of BN was defined as 0.6 µm, while the thickness of PyC was set to 0.03, 0.06, 0.12, and 0.25 µm. Both structural elements were subjected to the same compressive stress of 1000 Pa, and the elastic modulus and Poisson's ratio for BN and PyC were obtained from the software database.

## Conflict of Interest

The authors declare no conflict of interest.

## Author Contributions

M.L. conducted the experiments and wrote this manuscript; Q.G.F. designed and conceived this work; Y.P. and K.X.H. carried out the data acquisition and analysis; Y.D.Z. provided software and Data curation; X.H. discussed some questions and modified the manuscript.

## Supporting information



Supporting Information

Supporting MovieS1

## Data Availability

The data that support the findings of this study are available from the corresponding author upon reasonable request.
