# Supplementary material for: Armoring Boron Nitride with Pyrolytic Carbon Layers for Tunable Rigidity and Flexibility
Source: Adv Sci (Weinh). 2025 Jun 23;12(35):e04649. doi: 10.1002/advs.202504649 (PMC12463015; doi:10.1002/advs.202504649)
Supplement: Supplementary file 1 — Supporting Information [file ADVS-12-e04649-s002.docx]

**Supporting Information**

**Armoring Boron Nitride with Pyrolytic Carbon Layers for Tunable Rigidity and Flexibility**

Meng Lan^a^, Yu Pei^a^, Kexin Huang^a^, Yudong Zhou^b^, Xiao Han^a,^*, Qiangang Fu^a,^*

^a^ Shaanxi Key Laboratory of Fiber Reinforced Light Composite Materials, Science and Technology on Thermostructural Composite Materials Laboratory, Northwestern Polytechnical University, Xi’an, 710072, P. R. China

^b^ School of Medicine, Xi’an Jiaotong University, Xi’an, Shaan’xi Province, 710061, P. R. China

Corresponding author: xiao.han@nwpu.edu.cn (Xiao Han), fuqiangang@nwpu.edu.cn (Qiangang Fu)

**This supplement contains:**

Supplemental Fig. S1-S24

**
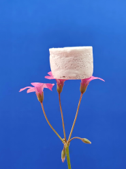
**

**Fig. S1.** Digital image of the lightweight BN aerogel placed on flower.


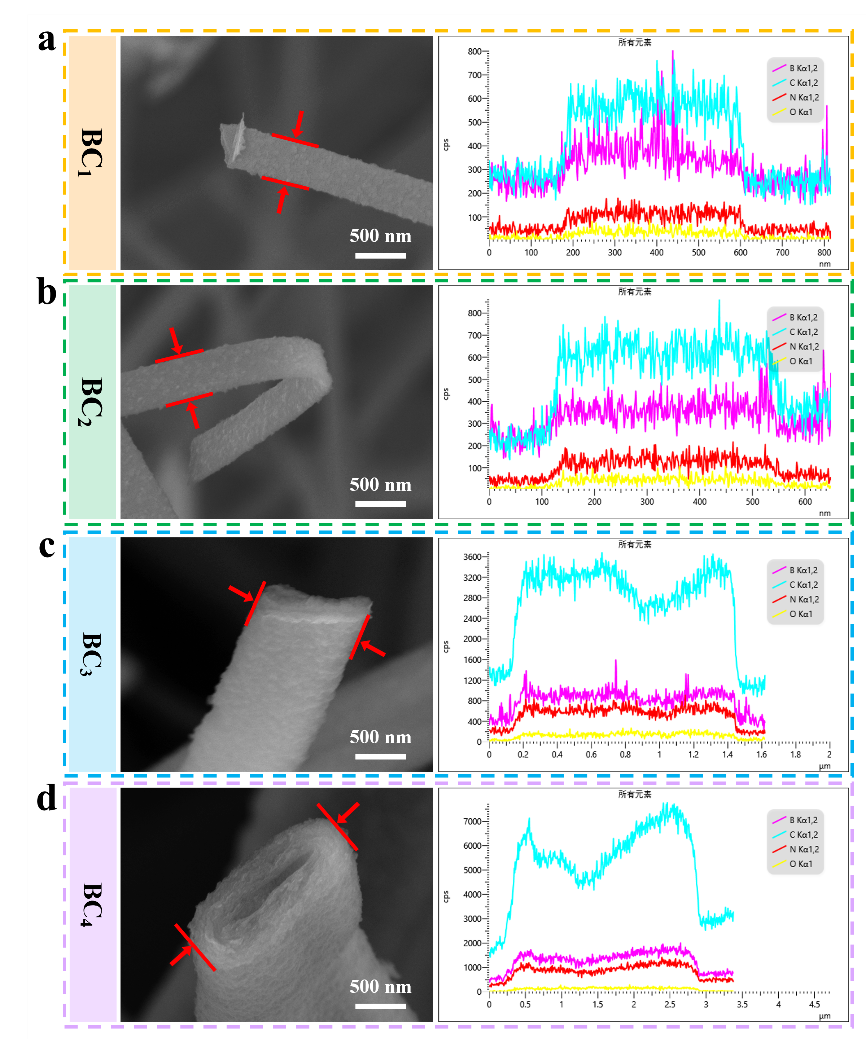


**Fig. S2.** (a-d) SEM images and Line scan element distribution curve of BC_x_ (x=1-4, the larger number represents a greater thickness of the depositing PyC layer).


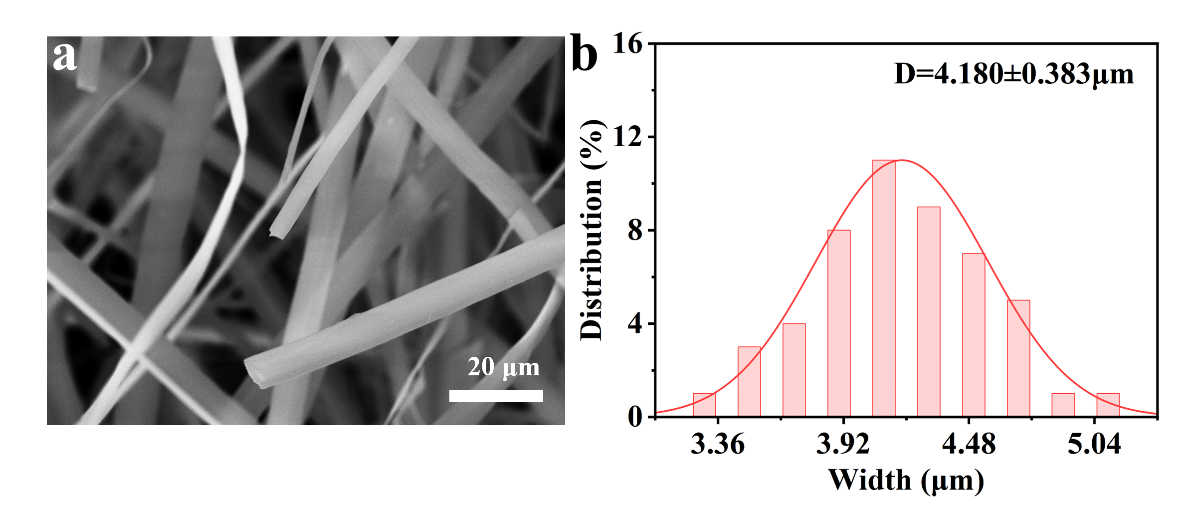


**Fig. S3.** (a) SEM image of BN aerogel. (b) The histogram distribution of the width of the BN ribbons.


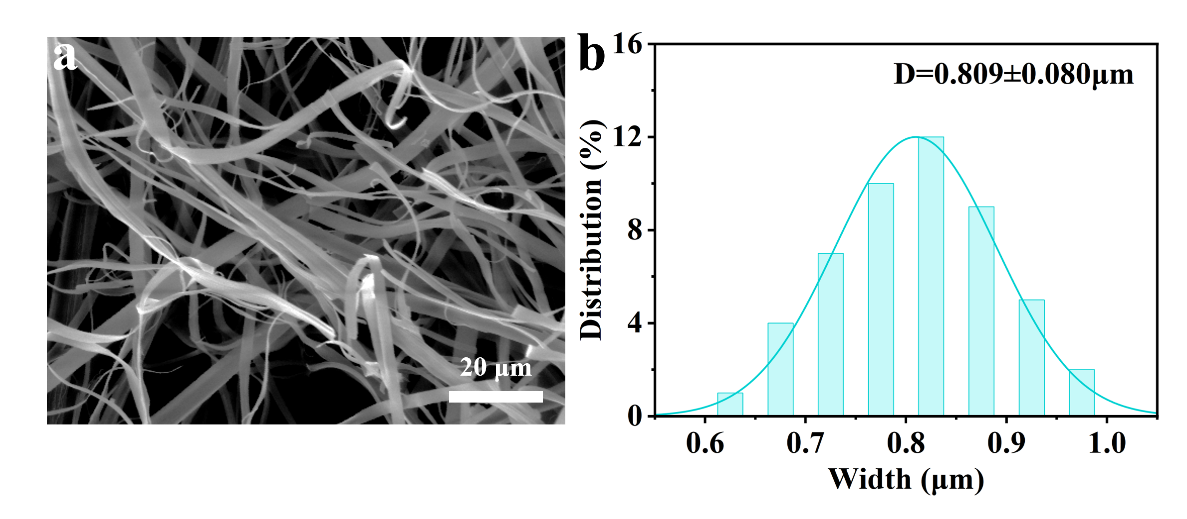


**Fig. S4.** (a) SEM image of BN aerogel after heat treatment at 1400°C in an argon atmosphere. (b) The histogram distribution of the width of the BN ribbons after heat treatment at 1400°C in an argon atmosphere.





**Fig. S5.** XRD patterns of original BN aerogel and BN aerogel after heat treatment at 1400°C in an argon atmosphere.





**Fig. S6.** Maximum stress, energy loss coefficient, and modulus versus compression cycles of BN at a density of 20 mg cm^-3^.





**Fig. S7.** Maximum stress, energy loss coefficient, and modulus versus compression cycles of BC_1_.





**Fig. S8.** Maximum stress, energy loss coefficient, and modulus versus compression cycles of BC_2_.





**Fig. S9.** Maximum stress, energy loss coefficient, and modulus versus compression cycles of BC_3_.





**Fig. S10.** Maximum stress, energy loss coefficient, and modulus versus compression cycles of BC_4_.


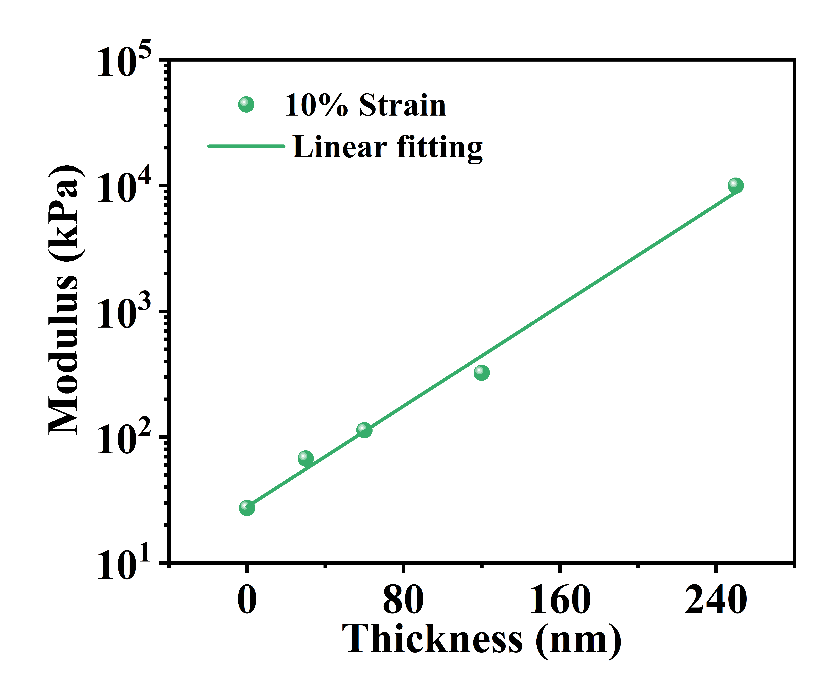


**Fig. S11.** Variation of modulus of sample with thickness of PyC.


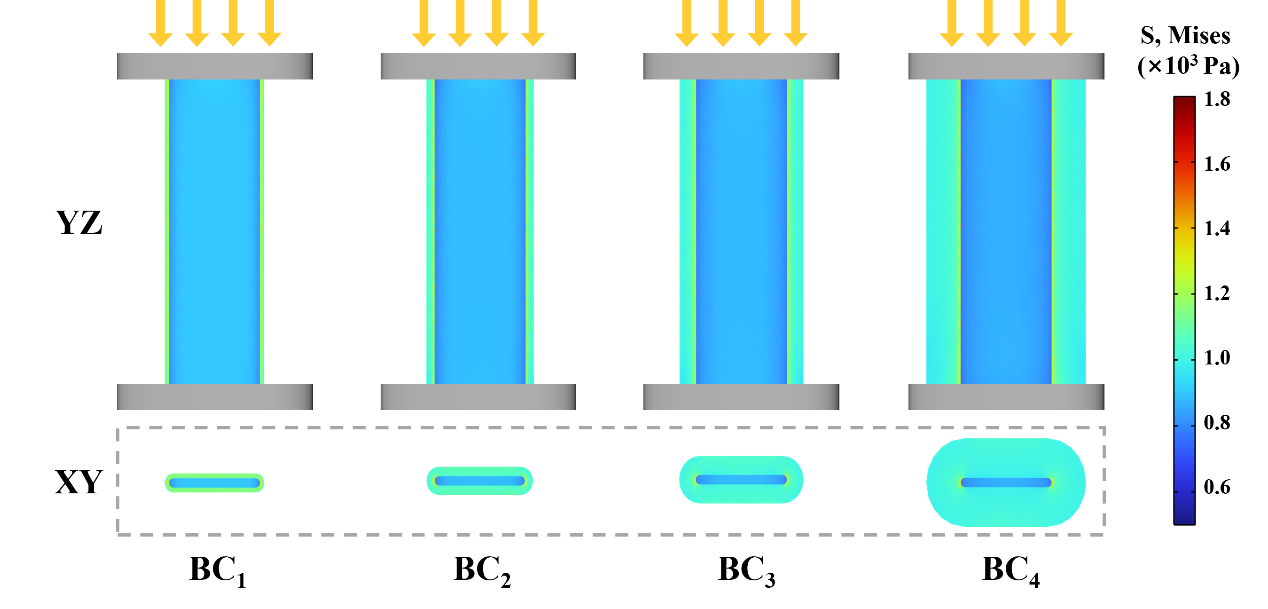


**Fig. S12.** Finite element simulation of stress distribution of nanoribbons with different PyC thicknesses during compression.


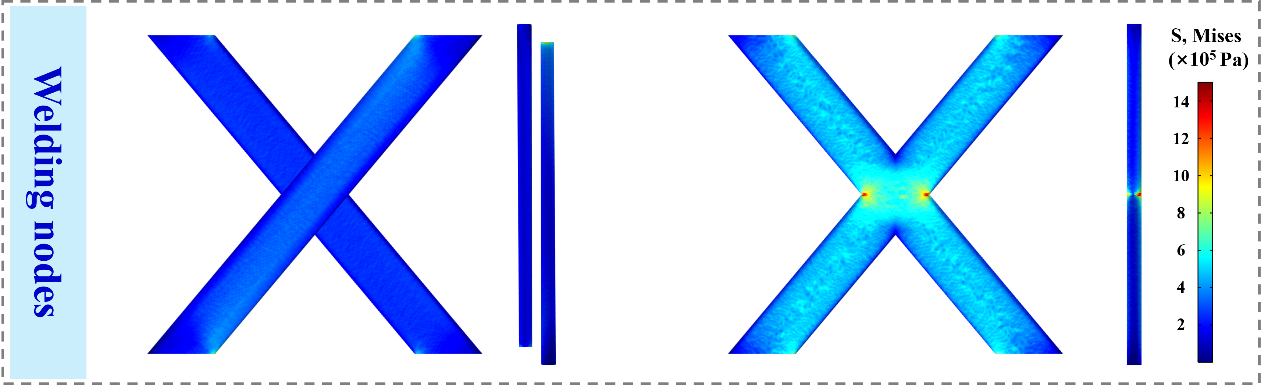


**Fig. S13.** Finite element simulation of stress distribution of nanoribbon networks with and without welding nodes during compression.


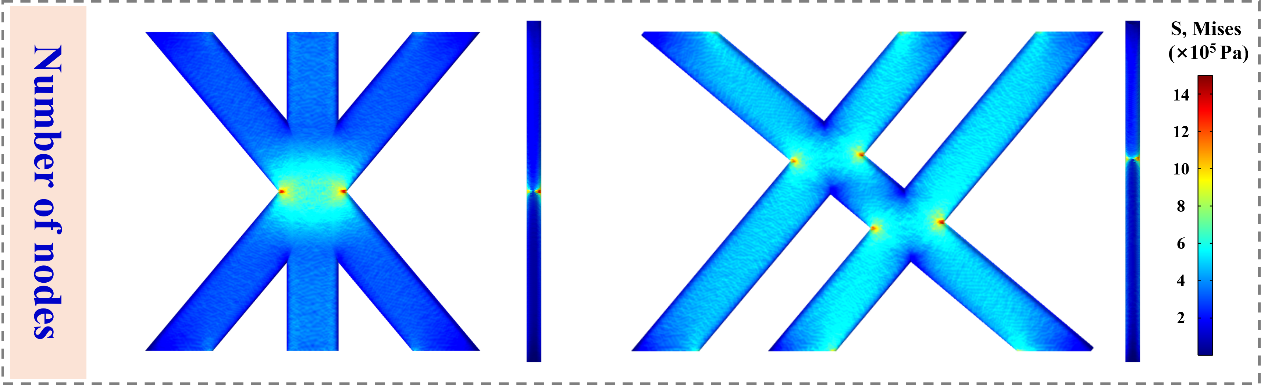


**Fig. S14.** Finite element simulation of stress distribution of nanoribbon networks with different numbers of welding nodes during compression.





**Fig. S15.** Maximum stress, energy loss coefficient, and modulus versus compression cycles of BN after heat treatment at 1400°C in an argon atmosphere.





**Fig. S16.** Maximum stress, energy loss coefficient, and modulus versus compression cycles of BC_1_ after heat treatment at 1400°C in an argon atmosphere.


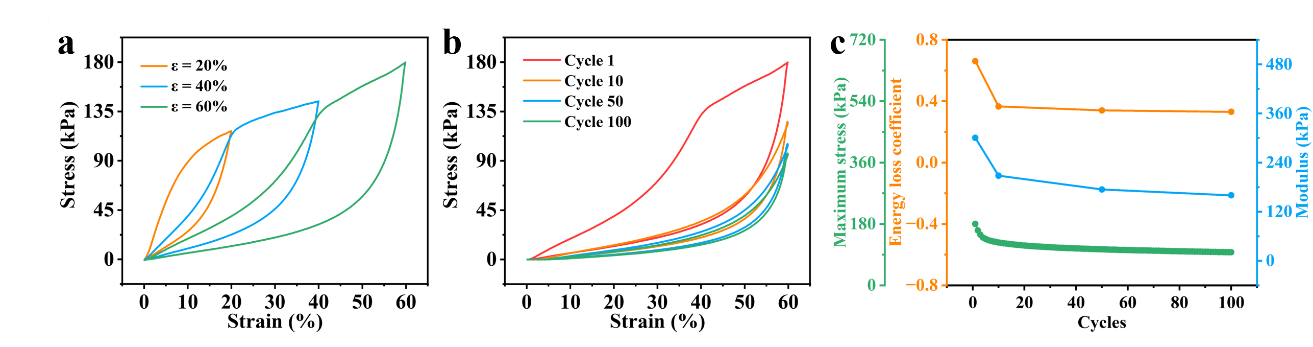


**Fig. S17.** (a) Compressive stress-strain curves and (b) compression fatigue tests of BC_3_ after heat treatment at 1400°C in an argon atmosphere. (c) Maximum stress, energy loss coefficient, and modulus versus compression cycles of BC_3_ after heat treatment at 1400°C in an argon atmosphere.





**Fig. S18.** Maximum stress, energy loss coefficient, and modulus versus compression cycles of BC_3_ after heat treatment at 1400 °C in an argon atmosphere.


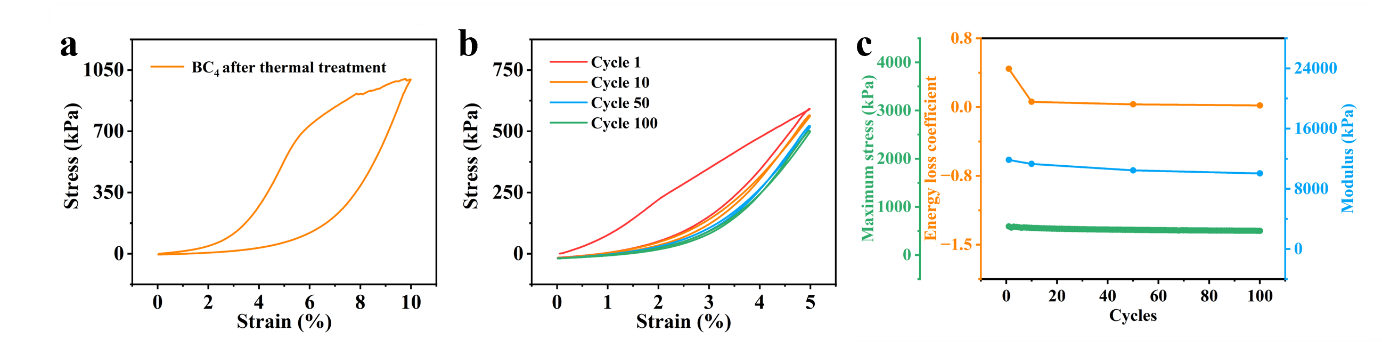


**Fig. S19.** (a) Compressive stress-strain curves and (b) compression fatigue tests of BC_4_ after heat treatment at 1400 °C in an argon atmosphere. (c) Maximum stress, energy loss coefficient, and modulus versus compression cycles of BC_4_ after heat treatment at 1400 °C in an argon atmosphere.





**Fig. S20.** Cross-section Temperature variation curves of BN and BN@PyC samples on a 120 °C heating stage.





**Fig. S21.** Trends in cross-section temperature as well as thermal conductivity of BN and BN@PyC samples.


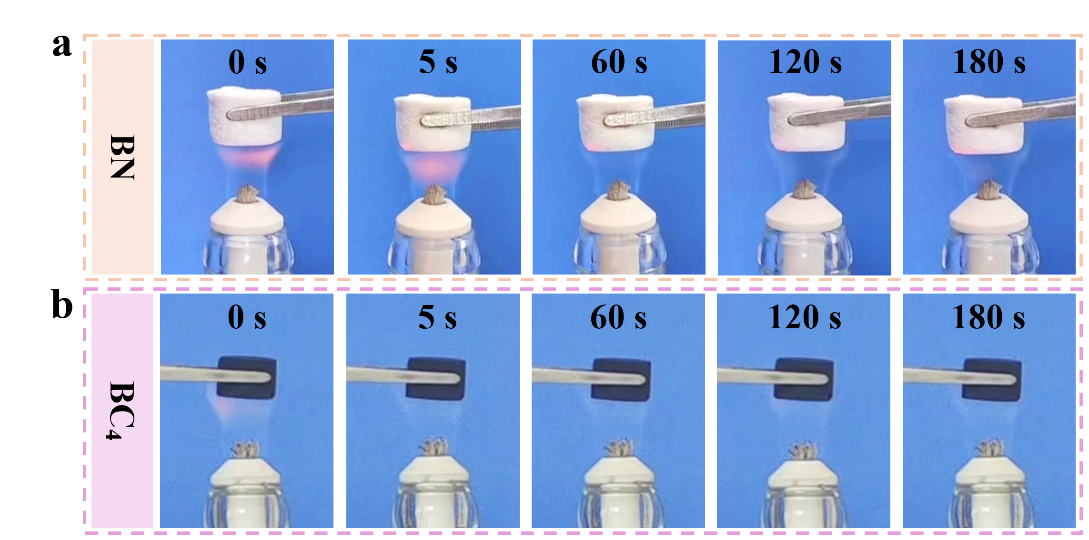


**Fig. S22.** The flame retardance of BN and BC_4_ samples.


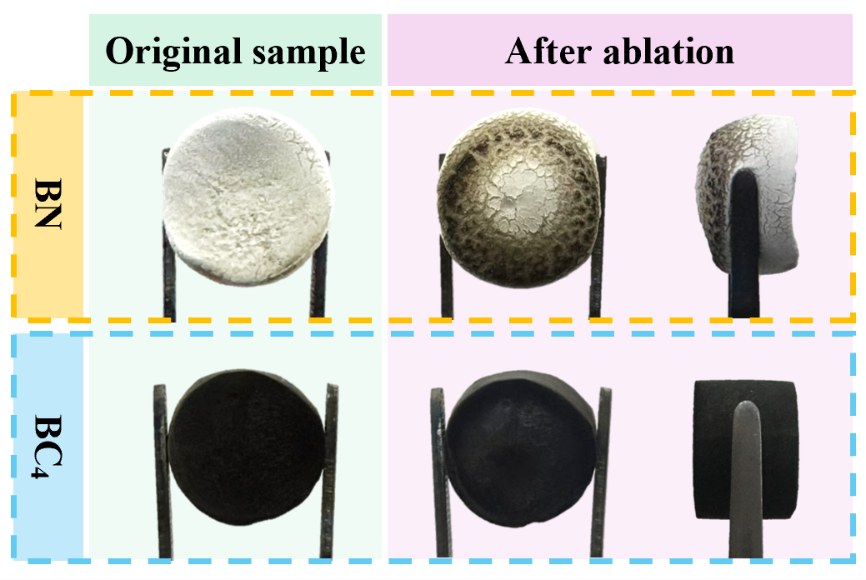


**Fig. S23.** Digital images of surface changes of BN and BC_4_ before and after ablation.


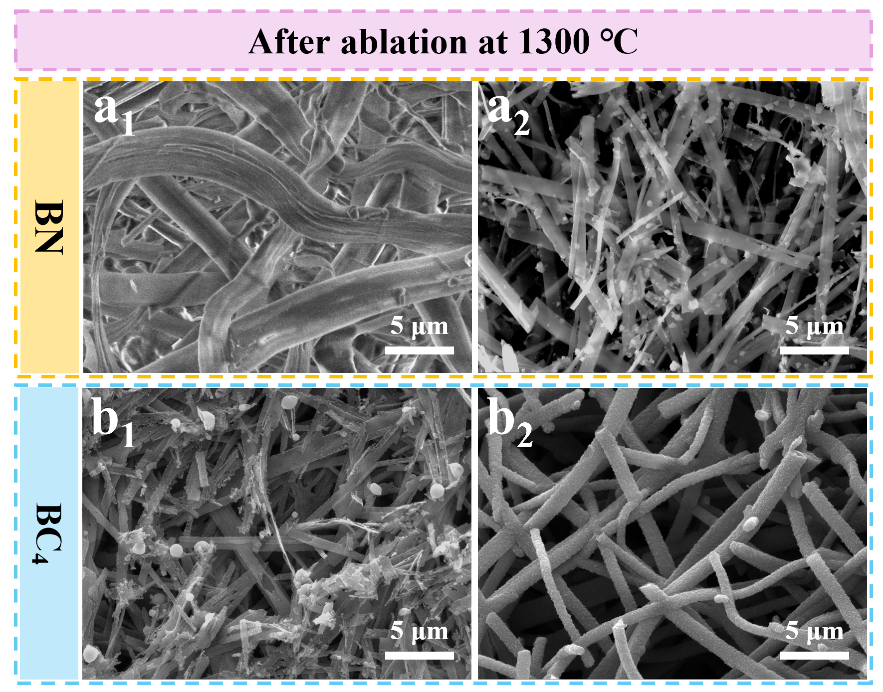


**Fig. S24**. SEM images of surface changes of BN and BC_4_ before and after ablation.
